# Factors Affecting the Physical Properties of Edible Composite Film Prepared from Zein and Wheat Gluten

**DOI:** 10.3390/molecules17043794

**Published:** 2012-03-27

**Authors:** Xingfeng Guo, Yanan Lu, Heping Cui, Xiangxing Jia, Hongchao Bai, Yuxiang Ma

**Affiliations:** School of Food Science and Technology, Henan University of Technology, Zhengzhou 450001, China; Email: guoxingfeng@haut.edu.cn (X.G.); yaya874276@163.com (Y.L.); cuihepingdavid@126.com (H.C.); jiaxiang8@126.com (X.J.); hsy1012@126.com (H.B.)

**Keywords:** zein, wheat gluten, edible composite film, properties

## Abstract

The effects of zein ratio, concentration of glycerol, liquid-solid ratio, ethanol concentration, pH and heat-treatment temperature on the properties of zein/wheat gluten composite films were researched. The results showed that elongation (E) increased with an increase in glycerol or ethanol concentrations, but it first increased and then decreased with increasing zein/wheat gluten ratio, heat-treatment temperature, pH and the ratio of liquid to solid; Tensile strength (TS) increased with the increase in heat-treatment temperature and pH, and decreased with the increase in glycerol or ethanol concentrations, and it reached a maximum value when the ratio of zein/wheat gluten was 20%, but had a minimum value when the ratio of liquid to solid was 8:1; Water Vapor Permeability (WVP) increased with an increase of glycerol concentration and the ratio of liquid to solid and ethanol concentration, but it decreased with increasing zein/wheat gluten ratio, heat treatment temperature, and pH of the film forming solution.

## Abbreviations

E: elongation
TS: Tensile strength
WVP: Water Vapor Permeability

## 1. Introduction

Increasing usage of non-degradable synthetic materials for food packaging has increased environmental pollution in the World, so considerable research effort has been devoted to the uses of edible films [1,2,3,4]. Proteins, such as soy, milk, and cereal proteins, are a class of renewable materials, and for technical applications such as films, coatings, adhesives, and disposables, the potential to use proteins has been recently recognized [5,6,7,8]. 

Zein is the most important protein in corn. It is a prolamin protein and therefore dissolves in 70%~80% ethanol. Zein is a relatively hydrophobic and thermoplastic material, and has excellent film forming properties, so it can be used for fabrication of biodegradable films. These film-forming properties have attracted attention in the field of edible film and coating materials. But the characteristic brittleness of zein diminishes its usefulness as a film, some modifications are needed to improve their flexibility [1,6,9,10]. In addition, preparation conditions also affect the properties of zein films [11,12].

Wheat gluten, which constitutes the protein by-product of starch preparation, is an interesting raw material for the development of biopolymers. Different processing methods including dry processing (thermoplastic process) and wet processing (solution casting), have been used to prepare wheat gluten films [3,6,13,14,15,16].

Recently, in order to improve the properties of protein films, many research groups have concentrated on the development of biodegradable polymer blends or composites made from corn gluten meal, wheat gluten, zein, soy protein, and other proteins [7,8,17,18,19]. 

The aim of preparing films from mixtures of structurally different proteins is to obtain composite materials in which each component provides a determined functional property [4,17]. Despite numerous studies on films of zein and gluten, respectively, there have been few reports on composite films of zein and gluten. In this paper, wheat gluten and zein were used as the main materials to prepare composite protein films. One of the outstanding features of wheat gluten among other proteins is its unique viscoelastic and flow properties which have already been subject to several investigations. Plasticized with glycerol gluten forms a malleable phase [14]. Although wheat gluten films may have unique cohesive and elastic properties, their very high water sensitivity and permeability was a hurdle to commercial exploitation [15]. Zein forms films with high tensile strength and low water vapor permeabilities compared to other protein-based films, and are too brittle for most applications [1,20]. The objectives of this paper were to prepare composite films based on zein and gluten, and to utilize effectively the functional merits of zein and gluten. The processing conditions that influence the characteristic of the films were also investigated.

## 2. Results and Discussion

### 2.1. Effect of Zein/Wheat Gluten Ratio on the Characteristics of the Composite Protein Films

**Figure 1:**
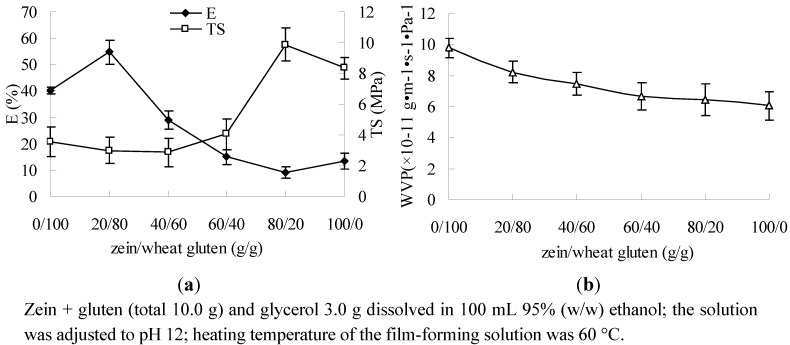
Effect of zein/wheat gluten ratio on E, TS(**a**) and water vapor permeability (WVP) (**b**).

The results showed that the films had maximum percentage elongation (E) when the ratio of zein/wheat gluten was 20/80 (g/g), and the tensile strength (TS) of the film was maximum when the ratio of zein was 80/20 (Figure 1a). Zein forms films with high TS compared to other protein-based films [20], and viscoelastic and flow properties are the outstanding features of wheat gluten among other proteins [14], it forms more elastic film. As the result, E of the composite film decreased and TS increased along with the increase of the zein/gluten ratio. 

Figure 1b shows that the water vapor permeability (WVP) decreased with the increase in the ratio of zein to wheat gluten. WVP is related to the properties of the protein. Gluten is a mixture of several proteins in which the glutelin molecules have more polar residues, on the other hand zein has more hydrophobic residues, so zein is a substantially better moisture barrier [6]. The films prepared from wheat gluten had higher WVP values than those prepared from zein, so the WVP decreased with the increasing ratio of zein content [21]. 

### 2.2. Effect of Concentration of Glycerol on the Characteristics of the Composite Protein Film

**Figure 2:**
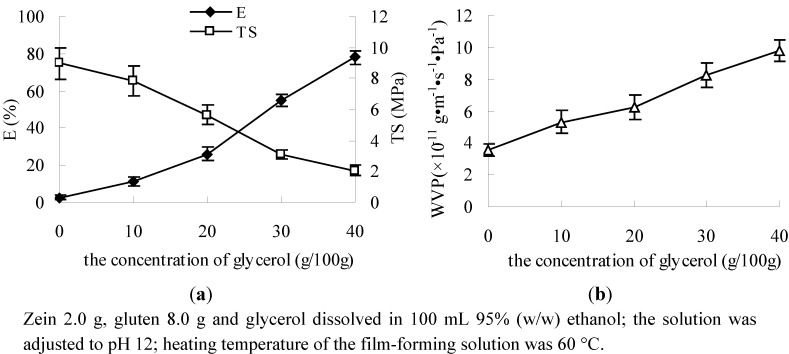
Effect of concentration of glycerol on E, TS (**a**) and WVP (**b**).

The results showed that E increased and TS decreased with an increase in the glycerol concentration (Figure 2a). This is possibly because unplasticized protein film has a low flexibility due to highly cooperative protein-protein interactions, mainly due to the high glutamine content which is responsible for numerous hydrogen bonds between the protein chains. Plasticization of edible films is believed to disrupt intermolecular interactions between polymer molecules with the effect of decreasing brittleness and increasing film flexibility. The plasticizing effect of polyols, particularly, can be attributed to their ability to locate between polymer molecules, bind water, and disrupt intermolecular polymer associations [1,22]. Glycerol is a small hydrophilic molecule which could be inserted between protein chains, hence acting as a plasticizer. With glycerol interspaced in the protein network, the distance between the protein chains was increased and direct interactions were reduced [23]. Moreover, the mechanical properties of the films were highly affected by the moisture content of the samples [24,25,26,27]. High levels of glycerol resulted in higher moisture content when different samples were conditioned under the same environment, the film with high moisture content exhibited high E and low E [24,28]. 

The WVP of the film increased along with the increase of concentration of glycerol (Figure 2b). This tendency could be explained by structural modifications of the protein network. The network may become less dense because of an increase in the mobility of the polymeric chains and in the free volume of the film [22,23,29]. Also, hygroscopic glycerol molecules increase the water content of the films and enhance the water holding capacity of the polymeric matrix, thus producing an increase in the effective diffusion coefficient of water vapor in the films, and water vapor transfer rates are closely related to the mobility of the polymer chain and free volume of chain segments [7,9,27,30].

### 2.3. Effect of the Heat-Treat Temperature on the Characteristics of the Composite Protein Film

**Figure 3:**
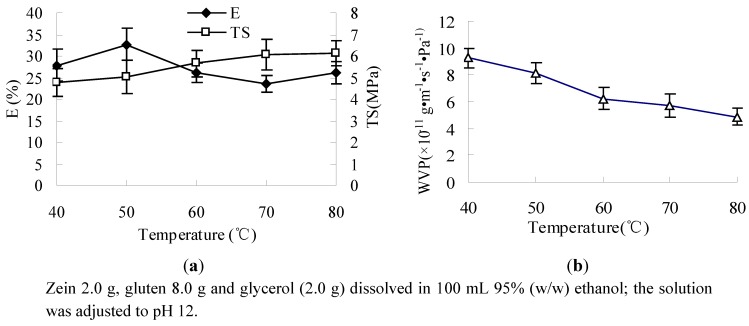
Effect of heat-treatment temperature on E, TS (**a**) and WVP (**b**).

The results showed that when the temperature was increased from 40 °C to 80 °C, the TS of the film was increased, and E first increased and then decreased (Figure 3a) and WVP decreased (Figure 3b). During heat treatment, parts of the three-dimensional structure of the proteins were unfolded, and parts of the hydrophobic residues, -SH groups, and S-S bonds, which were buried inside before heating, were exposed to water. When the molecular distances were close enough to each other, intermolecular polymerization occured through molecular forces of -SH, S-S interchange reaction, and/or hydrophobic bonds, resulting in an intermolecular network. As the result, after the film forming solution was heat-treated, TS of composite film was improved, and E was reduced [13,30].

The results showed that with the increase of the heating temperature of the film solutions, a lower WVP was observed (Figure 3b). This is possibly because at higher heating temperatures, greater cross-linking between protein-protein chains was formed, which resulted in a tight and compact protein network and structure. Thus the mobility of polypeptide chains was restricted, which reduced the diffusion of water molecules [13,16,30,31].

### 2.4. Effect of the Ratio of Liquid to Solid on the Properties of the Film

**Figure 4:**
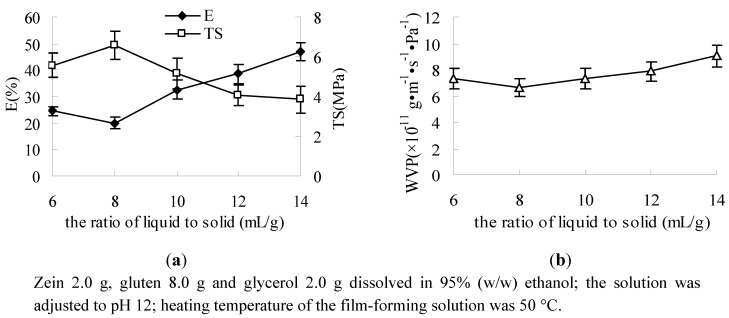
Effect of the ratio of liquid to solid on E, TS (**a**) and WVP (**b**).

The result showed that, at higher protein concentrations, the films became more resistant and presented a high TS; E decreased as protein concentration increased (Figure 4a). The mechanism to form a resistant film could involve a higher number and/or a better localization of bonds between protein chains. During the drying of the film-forming solution, ethanol and ammonium hydroxide were evaporated, allowing the formation of bonds between protein chains. During this stage, the proximity of protein chains induced by high protein concentrations resulted in such cross-bonds [23]. Because of the cross-bonds between protein molecules, the barrier properties of the film were improved and the WVP was decreased with the increase of protein concentration (Figure 4b).

### 2.5. Effect of Ethanol Concentration on the Properties of the Film

**Figure 5:**
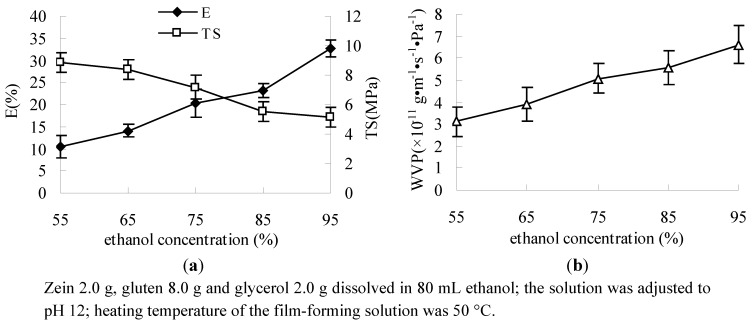
Effect of ethanol concentration on E, TS (**a**) and WVP (**b**).

The result showed that E of the film was increased and TS decreased along with the increase of ethanol concentration (Figure 5a). Glutenin had significant contribution on the TS of the film, on the other hand gliadin and zein had significant contribution on the E of the film. The solubility of gliadin and zein was increased along with the increase of ethanol concentration, so the E of the film increased and TS decreased [23,32]. With the increase of the ethanol concentration of the film-forming solution the WVP was improved (Figure 5b). At higher ethanol concentration the film became heterogeneous and this could explain the feasibility of water transmission [23].

### 2.6. Effect of pH on the Properties of the Film

**Figure 6:**
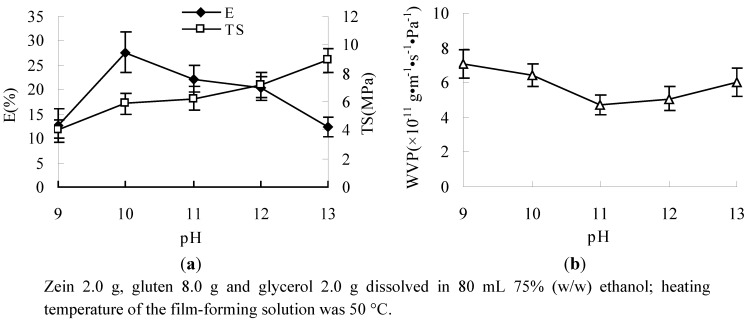
Effect of pH on E, TS (**a**) and WVP (**b**).

Effects of pH on mechanical properties are displayed in Figure 6a. The figures show that the E of the composite films first increased and then decreased with increasing the pH value of the film-solution from 9 to 13, and that the maximum values appeared at pH 10. At the same time the TS significantly increased as the pH increased. Under alkaline conditions, with the pH away from the isoelectric point, there is a promotion of the denaturation of proteins, unfolding and solubilization. When the pH of the film solutions was increased, SH/S-S interchange reactions or thiol/thiol (SH/SH) oxidations could occur upon heating and intermolecular disulfide (S-S) bonds formed [31].

Figure 6b shows that the WVP of the film first decreased and then increased with the increase of the pH of the film-solution. The WVP was the lowest at pH 11. When pH < 11, intermolecular disulfide (S-S) bonds could be formed, and the compacted network of the film was formed; but when pH > 11, the intermolecular electrostatic repulsion was strengthened and the structure of the film was relaxed, so WVP was increased.

## 3. Experimental

### 3.1. General

Wheat gluten (76.2% protein on dry weight base) was purchased from the market (Anhui Ante Biochemical Co. Ltd, Suzhou, China); zein was prepared in our lab (92.2% protein on dry weight base, N × 6.25) according to the method of Dickey *et al*. [33]. All other chemicals were analytical grade.

### 3.2. Preparation of Composite Film

According to the experimental design, the ratio of liquid to solid, zein/wheat ratio and glycerol concentration were changed when the film-forming solution was prepared. Zein was dissolved in ethanol; glycerol and wheat gluten were dissolved in another ethanol solution. The solutions were stirred, and anhydrous liquid ammonia (25%, w/w) was added into the wheat gluten solution to adjust the pH. The protein solutions were agitated and heated for 10 min each at setting temperature, and then combined, following a further agitation and heating at setting temperature for another 20 min. The sample was then cooled to 25 °C and centrifuged at 1,200 *g* for 5 min to remove the insoluble part. The composite films were formed by casting the mixed solutions onto leveled plastic plates (d = 12 cm) and then dried for 24 h at 50 °C. The formed films were peeled from the casting plates and held under controlled conditions (relative humidity 52%, temperature 25 °C) for 24 h prior to testing. 

### 3.3. Film Thickness

The film was cut into 5 cm × 1 cm strips. Film thickness was measured using a 0~2.5 mm micrometer screw gauge with overall thickness being expressed as an average (n = 10) taken randomly from each film. WVP and mechanical properties were calculated based on average thickness.

### 3.4. Film Tensile Strength (TS) and Elongation (E)

A TA.XT*plus* Texture Analyser (Stable Micro Systems, Surrey, UK) was used to analyze the mechanical properties in the tensile mode. Pretest speed = 10 mm/s, test speed = 3 mm/s, the initial grip length was 3 cm. The tensile strength (TS, MPa) was expressed as the force of film rupture (N) divided by the cross-sectional area of the film (m^2^), and the percentage elongation (E, %) was expressed as the tensile elongation of the film at rupture (mm) divided by the initial length of film (mm). The results for each sample were taken as the average of 10 measurements.

### 3.5. Water Vapor Permeability (WVP)

Water Vapor Permeability was determined according to the method of [34] with some modifications. The film was sealed on an aluminum permeation cup containing silica gel (0% RH) with silicone vacuum grease. The cup was placed at 30 °C in a desiccator containing the distilled water. It was weighed after 8 h. 6 Films were used for WVP testing. WVP of the film was calculated as follows:


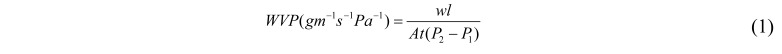


where *w* is the weight gain of the cup (g); *l* is the film thickness (m); *A* is the exposed area of film (m^2^); *t* is the time of gain (s); (*P*_2_ − *P*_1_) is the vapor pressure difference across the film (Pa). The results for of each sample were taken as the average of six measurements.

### 3.6. Statistical Analysis

The results were presented as mean values ± standard deviation. Significant differences among the samples were determined by ANOVA analysis.

## 4. Conclusions

The results showed that hydrophobicity of zein helps to reduce the WVP and lower ethanol concentration during the preparation of the films can also help to reduce the WVP of the films. The E of the film had a maximum value when the ratio of zein/wheat gluten was 20/80, and TS of the film was maximum when the ratio of zein was 80/20. The WVP decreased with the increase of zein concentrations. E increased and TS decreased along with the increase of the glycerol concentration; on the contrary, the WVP of the film increased with the increase of glycerol levels. When the temperature was increased from 40 °C to 80 °C, the TS of the film increased but the E first increased and then decreased. In addition, the WVP was reduced with the increase of the temperature. At higher protein concentrations, films became more resistant and showed a higher TS; at the same time a lower E and WVP were observed. With the increase of ethanol concentration, E and WVP of the film increased, but TS decreased. The E of the composite films first increased and then declined with increasing pH value of the film-forming solutions. The maximum E value appeared at pH 10, but the TS continually increased as the pH increased. The WVP of the film first decreased and then increased with the increase of the pH of the solutions. It was confirmed that combination of zein and gluten was suitable for film production.
